# Emerging non-thermal processing technologies and their impact on the quality, safety, and bioactive integrity of camel milk: A review article

**DOI:** 10.1016/j.fochx.2026.104459

**Published:** 2026-09-21

**Authors:** Shamsedin Mahdi Hassan, Tilahun Abera Teka, Samatar Abshir Mahamed, Maslah Mahamed Duale, Abdurazak Tahir Awale, Markos Makiso Urugo

**Affiliations:** a Jigjiga University, P.O. Box 1020, Jigjiga, Ethiopia; b Jimma University, P.O. Box 307, Jimma, Ethiopia; c Somali Region Livestock and Agricultural Research Institute, P.O. Box 198, Jigjiga, Ethiopia; d Addis Ababa Science and Technology University, Addis Ababa, Ethiopia; e University of Hargeisa, College of Agriculture, Veterinary Medicine and Environmental Studies, Hargeisa, Somalia; f Nutrition, Environmental Health, and Non-Communicable Disease Research Directorate, Ethiopian Public Health Institute (EPHI), P.O. Box 1242, Addis Ababa, Ethiopia

**Keywords:** Camel milk, Non-thermal processing, High-pressure processing, Pulsed electric fields, Ultrasonication, Microbial safety

## Abstract

Camel milk is a nutrient-rich functional food containing bioactive proteins, peptides, vitamins, minerals, and other constituents associated with antimicrobial, antioxidant, antidiabetic, and immunomodulatory activities. However, conventional thermal processing can compromise heat-sensitive nutrients and bioactive compounds. This review critically evaluates high-pressure processing (HPP), pulsed electric fields (PEF), ultrasonication, ultraviolet (UV-C), cold plasma, and ozonation regarding their effects on microbial safety, quality, and bioactive integrity of camel milk. A PRISMA-based literature search covering publications from 2001 to 2026 was conducted using relevant electronic databases, predefined search terms, and eligibility criteria, followed by systematic screening and qualitative synthesis of relevant studies. Evidence indicates that non-thermal technologies can achieve microbial reduction while limiting quality deterioration. HPP and ultrasonication have comparatively stronger camel-specific evidence, whereas evidence for PEF, UV-C, cold plasma, and ozonation remains limited. High costs, infrastructure constraints, cold-chain dependence, and insufficient camel-specific validation remain major barriers. Future research should prioritize standardized microbial inactivation kinetics, bioactive-retention studies, and techno-economic assessment to support safe and scalable application.

## 1 Introduction

Camels, often referred to as the “ships of the desert,” are crucial to the survival of pastoralist societies in arid and semi-arid regions (Sazmand & Nourian, 2023). These animals have evolved to withstand severe environmental conditions, including prolonged exposure to high temperatures and water scarcity, making them an essential livestock resource in regions where other species may struggle to thrive (Kandeel et al., 2022). In recent years, the global camel population has increased steadily, particularly in populations reared for milk production. This growth has been accompanied by increasing demand for camel milk, driven by its distinctive nutritional composition and reported health-promoting properties (Ait El Alia et al., 2025; Hassan et al., 2026; Lajnaf et al., 2022).

Despite its nutritional and functional significance, maintaining the quality, safety, and bioactive integrity of camel milk during processing remains challenging. Conventional thermal processing is effective for microbial inactivation but may induce undesirable changes in heat-sensitive proteins, enzymes, vitamins, and other bioactive constituents (Soni et al., 2021). Consequently, increasing attention has been directed toward alternative preservation strategies capable of achieving microbial safety while minimizing processing-induced quality losses. Non-thermal processing technologies have emerged as promising alternatives because they can reduce microbial loads under conditions that may limit the thermal degradation of sensitive milk constituents, thereby helping to preserve fresh-like characteristics and extend shelf life (Pegu & Arya, 2023; Radhakrishnan et al., 2023). However, their effectiveness depends strongly on processing intensity and treatment conditions, and preservation of quality cannot be assumed solely from the designation of a technology as “non-thermal.”

The growing consumer preference for minimally processed and natural foods has further increased interest in these technologies within the dairy sector (Hassan et al., 2026). This demand creates a technological need for preservation strategies that can simultaneously provide adequate microbial safety and minimize chemical, structural, nutritional, and sensory changes associated with processing. This consideration is particularly important for camel milk because its distinctive protein profile, endogenous enzymes, minerals, vitamins, and bioactive constituents may respond differently to processing conditions than those of conventional bovine milk. Accordingly, findings from bovine and other dairy systems can provide useful mechanistic and technological context, but they should not be assumed to represent equivalent responses in camel milk.

Non-thermal processing may facilitate the retention of valuable constituents such as immunoglobulins, lactoferrin, lysozyme, vitamins, and other bioactive compounds associated with the nutritional and functional value of camel milk (Rakhmatulina et al., 2024). However, the relationship between microbial inactivation and preservation of these constituents is complex. Processing parameters such as pressure, electric-field strength, treatment duration, acoustic intensity, ultraviolet dose, plasma characteristics, and ozone exposure can determine both microbial lethality and chemical or biochemical changes in the milk matrix. For example, treatment intensity may influence protein unfolding and aggregation, enzyme inactivation, oxidation of lipids and vitamins, modification of bioactive peptides, and redistribution or altered availability of minerals. Thus, assessment of non-thermal processing requires consideration not only of microbial reduction but also of the molecular mechanisms governing changes in nutritional and bioactive quality.

Beyond quality preservation, some non-thermal technologies may provide advantages in energy use, processing efficiency, and reduction of chemical preservatives, potentially supporting more sustainable food-processing systems (Neokleous et al., 2022). Nevertheless, their industrial relevance depends on a balance among microbial efficacy, retention of desirable constituents, processing cost, throughput, scalability, packaging requirements, shelf-life, regulatory acceptance, and control of post-treatment contamination. These considerations are particularly important for camel milk, where the evidence base remains less extensive than that available for conventional dairy systems.

Previous reviews have addressed aspects of camel milk composition, nutritional properties, processing, and emerging preservation technologies. However, important gaps remain in the critical integration of processing conditions with the chemical and biochemical responses of camel milk. Existing literature also contains substantial variation in reported outcomes, with differences attributable to treatment intensity, processing duration, milk composition, microbial load, analytical approaches, and experimental design (Alhaj et al., 2022; Arain et al., 2024; Mazumder et al., 2024; Mohamed et al., 2022). Moreover, camel-specific evidence is frequently considered alongside findings from bovine and other dairy systems, making it difficult to determine which effects have been directly demonstrated in camel milk and which represent evidence transferred from other dairy matrices. A critical synthesis that distinguishes these evidence bases and examines their consistency is therefore needed.

In this context, the present review critically examines emerging non-thermal processing technologies for camel milk, with particular emphasis on the mechanisms governing microbial inactivation and processing-induced changes in proteins, enzymes, minerals, vitamins, lipids, and other bioactive constituents. HPP, PEF, ultrasonication, cold plasma, ultraviolet treatment, ozonation, and related technologies are evaluated in relation to their processing conditions, microbial efficacy, quality retention, and technological limitations. Particular attention is given to conflicting findings, treatment-dependent responses, and the extent to which reported benefits are supported by direct camel milk evidence. The review also considers the implications of these findings for industrial application, including scalability, process validation, safety assurance, sustainability, and the identification of research priorities.

By integrating the technological, microbiological, chemical, and biochemical dimensions of non-thermal processing, this review provides a critical assessment of how emerging preservation approaches can be applied to camel milk while maintaining its nutritional and bioactive integrity. It further highlights the limitations of the current evidence base and identifies areas requiring targeted investigation to support the development of safe, effective, and commercially viable non-thermal processing strategies for camel milk.

## 2 Methodology

A PRISMA-based structured literature search was conducted to identify studies on emerging non-thermal processing technologies and their effects on the quality, safety, nutritional composition, and bioactive integrity of camel milk. Relevant literature was searched in Scopus, Web of Science, PubMed, and ScienceDirect using combinations of keywords related to camel milk, non-thermal processing, microbial safety, quality, proteins, enzymes, minerals, vitamins, and bioactive compounds, covering publications from 2001 to 2026. Reference lists of relevant articles and reviews were additionally screened to identify further eligible studies. Studies were included when they evaluated non-thermal processing of camel milk or provided relevant mechanistic or comparative evidence from other dairy systems; unrelated studies, duplicates, and publications lacking sufficient relevant information were excluded.

The literature selection process followed the PRISMA sequence of identification, duplicate removal, title-and-abstract screening, full-text assessment, and final inclusion against predefined eligibility criteria. A total of 336 records were initially identified through the database search. After 42 duplicate records were removed, 294 unique records remained for title-and-abstract screening, of which 108 were excluded for falling outside the scope of the review (not related to camel milk or non-thermal processing, wrong publication type, or non-English language). The remaining 186 records underwent full-text assessment, and a further 61 were excluded, primarily for insufficient camel-milk-relevant data, duplicate or overlapping datasets, or failure to meet peer-review or data-reporting standards. This process yielded a final set of 125 sources comprising original camel-milk experimental studies, comparative or mechanistic studies in other dairy systems, and review articles that were included in the qualitative synthesis and cited throughout this review. Fig. 1 presents the corresponding PRISMA flow diagram, showing the numbers of records identified, screened, excluded, and included. During evidence synthesis, camel-milk-specific experimental studies were distinguished from studies conducted in bovine or other dairy systems, and review articles were distinguished from primary research. Extracted information included processing technology and conditions, microbial targets and reductions, milk composition, effects on proteins, enzymes, minerals, vitamins and bioactive compounds, analytical methods, and statistical significance where reported.

**Fig. 1:**
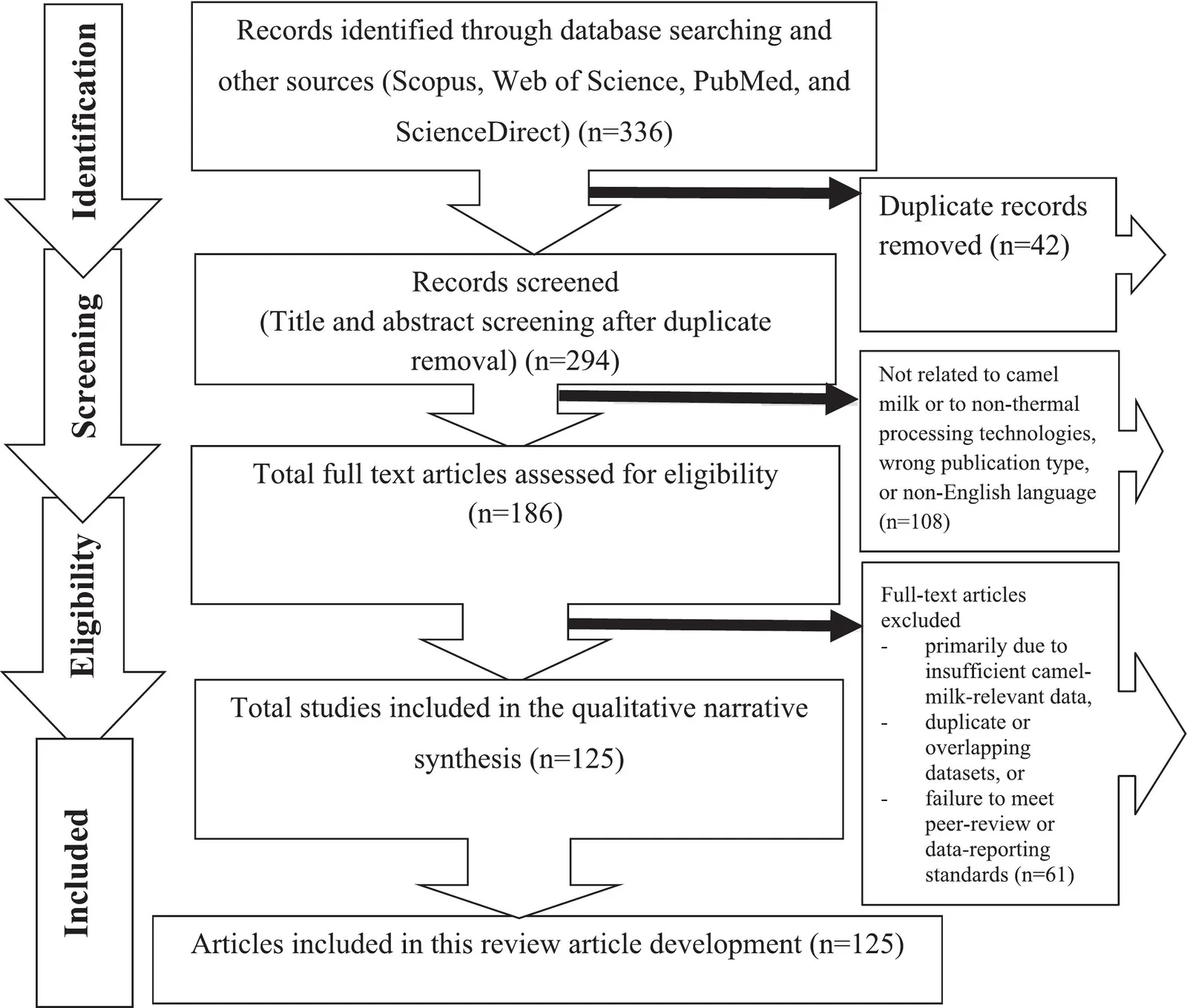
PRISMA–based flow diagram illustrating the literature search, screening, and study.

Evidence was synthesized critically and comparatively rather than on a purely study-by-study basis, with particular attention to conflicting findings, treatment-dependent responses, dose–response relationships, and differences in microbial inactivation and in the retention of nutritional and bioactive components. Where comparable evidence was available, technologies were evaluated in relation to microbial efficacy, quality and bioactive retention, processing requirements, cost, throughput, scalability, and industrial readiness. Evidence from other dairy systems was used primarily to provide mechanistic or comparative context where camel-specific evidence was limited, and was explicitly distinguished from direct camel-milk evidence throughout. Differences in processing conditions, analytical methods, and the availability of camel-specific studies were taken into account when interpreting findings and drawing conclusions.

## 3 Properties of camel Milk processing: Heat sensitivity, protein structure, enzyme activity, and processing implications

Camel milk is increasingly recognized for its distinctive nutritional and functional properties; however, its processing presents significant challenges because of its unique protein composition, enzyme systems, and thermal behavior compared with bovine milk. The absence of *β*-lactoglobulin, relatively low κ-casein content, high proportion of β-casein, and differences in casein micelle structure contribute to its distinctive processing characteristics and may affect its stability during conventional dairy processing (Abduku & Eshetu, 2024; Arain et al., 2023; Hailu et al., 2016; Ho et al., 2022; Seifu, 2023). Heat treatment can induce protein unfolding, denaturation, aggregation, and degradation of heat-sensitive bioactive constituents, with the magnitude of these changes depending on temperature and treatment duration (Benabdelkamel et al., 2017; Gebrehiwot & Banat, 2025; He et al., 2025; Li et al., 2020).

**These changes are not simply compositional losses; they involve alterations in protein conformation, intermolecular interactions, surface hydrophobicity, and aggregation behavior that can subsequently influence solubility, functionality, digestibility, and bioactivity.** Endogenous enzyme systems, particularly proteolytic enzymes such as plasminogen/plasmin, further influence the stability, texture, and quality of processed camel milk products (Mbye et al., 2021; Mbye et al., 2022). Non-thermal technologies, including high-pressure processing and ultrasonication, are therefore being investigated as alternatives that may achieve microbial safety while limiting undesirable thermal and chemical changes (Mazumder et al., 2024; Mazumder et al., 2026; Mudgil et al., 2022). **Importantly, the extent of preservation is technology- and treatment-dependent, and the term “non-thermal” should not be interpreted as an assurance that structural or biochemical changes will be absent.**

### 3.1 Heat sensitivity of camel Milk proteins

Camel milk proteins exhibit distinctive thermal stability profiles compared with bovine milk. Caseins in camel milk can undergo substantial structural changes during heating, while whey proteins, although relatively more resistant under moderate conditions, also undergo denaturation at elevated temperatures (Benabdelkamel et al., 2017; Gebrehiwot & Banat, 2025; He et al., 2025; Li et al., 2020). Heating above 105 °C has been associated with the formation of large protein aggregates, driven partly by hydrophobic interactions, with consequent effects on β-casein stability and protein functionality (He et al., 2025). The absence of β-lactoglobulin, a major whey protein in bovine milk, also results in a different pattern of heat-induced protein interactions and aggregation in camel milk (Laleye et al., 2008; Maryniak et al., 2023).

**At the molecular level, thermal treatment disrupts non-covalent interactions that maintain native protein conformation. Unfolding exposes previously buried hydrophobic and reactive groups, promoting protein-protein interactions and, under sufficiently severe conditions, the formation of larger aggregates. These changes can modify solubility, emulsifying properties, gelation behavior, and the accessibility of proteolytic or bioactive sites. Consequently, the technological effect of heating depends not only on the final temperature but also on the heating rate, holding time, protein concentration, and composition of the milk matrix. Evidence from bovine milk provides useful mechanistic comparisons, but the distinct casein and whey protein composition of camel milk warrants cautious extrapolation.**

### 3.2 Protein structure changes during processing

Heat treatment can cause unfolding and denaturation of camel milk proteins, reflected by changes in secondary and tertiary structural organization, including reductions in α-helical structures and alterations in β-sheet, β-turn, and random-coil arrangements (Benabdelkamel et al., 2017; He et al., 2025; Li et al., 2020). **These conformational changes can increase surface hydrophobicity and expose reactive amino acid residues, facilitating aggregation and potentially altering the biological activity and functionality of proteins. The consequences therefore extend beyond simple denaturation and may include changes in protein solubility, digestibility, intermolecular interactions, and the accessibility of bioactive domains (****Liu et al.,** 2022**;** Manzoor **et al.,** 2026**;** **Rahaman et al.,** 2015**;** **Saricay et al.,** 2014**;** **Tang et al.,** 2021**).** Heat-sensitive proteins such as lactoferrin and immunoglobulins may be particularly susceptible to processing-induced structural modification, although the extent of change depends strongly on treatment severity and the specific analytical method used (Goulding et al., 2021; Korhonen & Marnila, 2009; Wang et al., 2019).

Severe thermal processing can also promote chemical reactions involving proteins and reducing sugars. Spray drying at high inlet temperatures (>170 °C) has been associated with increased formation of Maillard reaction products and reduced nutritional quality, particularly through modification and degradation of lysine residues (Gebrehiwot & Banat, 2025; Perusko et al., 2021). **This mechanism is particularly relevant to camel milk because lactose provides a reducing sugar capable of participating in Maillard chemistry with protein amino groups. Xiang et al. (2026) provides recent mechanistic context for lactose-related Maillard reactions, demonstrating how advanced glycation and related reaction pathways can contribute to chemical modification of proteins and deterioration of nutritional quality. Thus, under severe thermal conditions, protein damage may involve not only physical unfolding and aggregation but also irreversible chemical modification of amino acid residues. Such reactions provide an additional chemical rationale for investigating processing strategies that achieve microbial safety under less severe thermal conditions.**

**The extent of these changes should nevertheless be interpreted in relation to the actual processing conditions. High-temperature short-time treatments, prolonged heating, and severe drying conditions may produce different degrees and types of modification, and findings from one processing configuration should not automatically be generalized to all thermal treatments (****Alhaj et al.,** 2026**; Y.** **Wang et al.,** 2024**;** van Lieshout et al., 2020**). Freeze-drying generally provides better preservation of protein profiles because of the lower thermal exposure, although its relatively high cost and limited throughput can constrain industrial application (****Zou et al.,** 2022**).**

### 3.3 Enzyme activity effects

Endogenous enzymes are important determinants of camel milk stability and processing behavior. Proteolytic systems, particularly plasminogen/plasmin, can remain active during cheese and yogurt production and contribute to extensive proteolysis, influencing protein breakdown and the softer texture commonly reported for camel milk products compared with their bovine counterparts (Mbye et al., 2021; Mbye et al., 2022). **From a biochemical perspective, enzyme inactivation is governed by both the intrinsic thermal stability of the enzyme and the processing history. Progressive heating can alter the enzyme's tertiary structure and active-site conformation, reducing catalytic activity; however, the rate of inactivation is not necessarily identical among enzymes. Consequently, a single processing temperature cannot be assumed to produce equivalent inactivation of all endogenous enzymes (****Deeth,** 2021**;** **Dickow et al.,** 2012**;** **Makroo et al.,** 2020**).**

Lactoperoxidase is relatively heat-sensitive, with substantial activity losses reported at temperatures associated with pasteurization and more severe heating (Zou et al., 2022). In contrast, alkaline phosphatase exhibits comparatively greater thermal resistance in camel milk than in bovine milk, although severe heating can eventually reduce its activity (Tzereme et al., 2025). **These differences demonstrate why enzyme activity can serve both as an indicator of processing severity and as an important quality attribute in its own right. However, enzyme inactivation should not be treated as synonymous with microbial inactivation because enzymes and microorganisms can exhibit markedly different thermal resistance profiles.**

**Overall, the thermal sensitivity of camel milk reflects the combined effects of protein composition, molecular structure, endogenous enzyme systems, and chemical reactions such as Maillard chemistry. These characteristics provide an important basis for evaluating emerging non-thermal technologies. Rather than assuming that reduced thermal exposure automatically preserves all quality attributes, subsequent sections therefore examine how individual technologies and their processing parameters influence microbial safety, protein structure, enzyme activity, and the retention of nutritional and bioactive constituents (He et al., 2026;** Laleye et al., 2008; Mohamed et al., 2022;**).**

## 4 Processing implications & novel technologies

Traditional thermal processing can adversely affect the technological functionality of camel milk, including rennetability, gel strength, sensory attributes, and nutritional quality, particularly when processing conditions promote extensive protein denaturation and aggregation (Arain et al., 2023; Genene et al., 2019; Seifu, 2023). These effects are particularly relevant for camel milk because processing conditions optimized for bovine milk cannot necessarily be transferred directly to camel milk. The technological challenge is therefore to establish treatment conditions that achieve the required microbial safety while minimizing changes in protein functionality, bioactive integrity, sensory quality, and other desirable characteristics (Abou-Soliman et al., 2025; Arain et al., 2024; Mohamed et al., 2022; Seifu, 2023). Emerging non-thermal technologies, including HPP, ultrasonication, PEF, cold plasma, ultraviolet treatment, and ozonation, provide alternative approaches, but their technological performance varies considerably according to treatment intensity, exposure time, milk composition, target microorganisms, and the quality attribute being evaluated (Al-Thaibani et al., 2024; Mazumder et al., 2026; Mbye et al., 2023; Mudgil et al., 2022; Sobti et al., 2024).

HPP has received comparatively greater attention in camel milk and currently provides some of the more substantial evidence among emerging non-thermal technologies. Its principal advantage is the ability to achieve substantial microbial inactivation at pressures commonly reported within approximately 400–600 MPa while limiting the direct thermal load. However, the outcome is not pressure-dependent alone; treatment duration, temperature, initial microbial load, and the resistance characteristics of the target organism influence the magnitude of microbial reduction (EFSA Panel on Biological Hazards (BIOHAZ Panel) et al., 2022; Elsahoryi et al., 2026; Mazumder et al., 2024; Osaili et al., 2025). HPP can also induce pressure-dependent protein conformational changes, meaning that microbial efficacy and preservation of functional properties must be considered simultaneously (Alhaj et al., 2026; Serna-Hernandez et al., 2022; Siddiqui et al., 2024). Thus, claims of pasteurization-equivalent safety or superior quality retention should be interpreted in relation to the specific microorganisms, pressure-time conditions, and analytical endpoints evaluated rather than generalized across all HPP applications.

Ultrasonication similarly demonstrates potential for microbial control and preservation of selected quality attributes, but its effectiveness is strongly dependent on acoustic intensity, frequency, treatment duration, temperature development, and matrix characteristics. Reported microbial reductions should therefore be interpreted together with the initial microbial load, target organism, and detection method (El Kindoussy et al., 2026; Guimarães et al., 2021; Mudgil et al., 2022). In particular, reports of complete inactivation based solely on culture-dependent non-detection require caution because injured or viable-but-nonculturable cells may remain undetected. Moreover, direct ultrasonication may present challenges for scale-up because treatment uniformity and energy transfer can become more difficult to control as processing volume increases (Mudgil et al., 2022). Accordingly, the available evidence supports ultrasonication as a promising processing approach, but its industrial readiness should not be inferred solely from laboratory-scale microbial reductions.

Evidence for PEF, cold plasma, ultraviolet treatment, and ozonation in camel milk remains comparatively limited. PEF has demonstrated considerable potential in other dairy systems because electroporation can disrupt microbial cell membranes under appropriately controlled electric-field conditions; however, direct camel-milk experimental evidence remains insufficient to establish its performance across relevant microorganisms and processing conditions (Nabilah et al., 2022; Siddique et al., 2025; Urugo et al., 2023; Vashisht et al., 2024). Consequently, PEF should currently be regarded as a promising technology with potential applicability rather than a technology whose industrial performance in camel milk has been fully demonstrated. Similarly, cold plasma, ultraviolet treatment, and ozonation may provide effective microbial control, but their application to camel milk requires greater consideration of matrix effects and treatment-specific quality changes. In particular, reactive oxygen and nitrogen species generated during plasma treatment and ozone exposure may promote lipid oxidation or modify proteins, vitamins, and sensory attributes if treatment intensity is excessive (Bayati et al., 2026; Botondi et al., 2023; Dhahir et al., 2021; Keewan et al., 2026; Nikmaram & Keener, 2022). These potential quality trade-offs require systematic evaluation alongside microbial efficacy.

Comparative evidence therefore does not support a universal ranking of non-thermal technologies based solely on microbial inactivation. HPP currently has a relatively stronger camel-milk evidence base, whereas PEF, cold plasma, ultraviolet treatment, and ozonation require further camel-specific validation (Elsahoryi et al., 2026; Kateh et al., 2024; Mazumder et al., 2024). Across technologies, industrial potential should instead be assessed using multiple criteria, including microbial reduction, retention of nutritional and bioactive components, effects on physicochemical and sensory quality, processing cost, throughput, energy requirements, scalability, process validation, packaging requirements, shelf-life, and regulatory acceptance. Technologies that perform well at laboratory scale may not necessarily provide equivalent advantages under continuous or industrial processing conditions.

An additional consideration is the distinction between preservation of individual constituents and preservation of overall product functionality. A treatment may retain a high proportion of a particular bioactive protein while simultaneously modifying other proteins, enzymes, lipids, or sensory attributes. Similarly, microbial inactivation achieved under severe processing conditions may be accompanied by undesirable chemical changes. Therefore, technological optimization should target an appropriate balance between microbial safety and the preservation of the broader chemical and functional characteristics of camel milk rather than maximizing a single processing outcome.

Despite advances in understanding camel milk processing, important knowledge gaps remain regarding technology-specific dose-response relationships, organism-specific microbial reductions, treatment thresholds for quality deterioration, and the comparability of outcomes across processing platforms. Direct comparative studies using standardized camel milk, defined microbial loads, harmonized analytical methods, and conventional pasteurization controls are particularly needed. Such studies would enable more reliable determination of whether the advantages observed for individual non-thermal technologies translate into meaningful improvements over conventional processing. Future research should therefore prioritize process optimization based on camel milk-specific characteristics rather than direct adoption of bovine milk protocols, while also addressing scale-up, process validation, post-treatment contamination, shelf-life, and economic feasibility.

## 5 Non-thermal processing technologies for camel milk

Thermal treatment remains the conventional approach for ensuring the microbiological safety and extending the shelf life of milk. Pasteurization effectively reduces pathogenic and spoilage microorganisms, but the associated thermal load can induce protein denaturation, aggregation, enzyme inactivation, vitamin degradation, Maillard reactions, and sensory changes, depending on the temperature–time combination (Chemat & Khan, 2011; Fox et al., 2015; Shori, 2015). These effects are particularly relevant for camel milk because its distinctive protein composition and bioactive profile can result in processing responses that differ from those of bovine milk, and because camel milk's high perishability makes effective preservation essential for its wider commercialization (Mazumder et al., 2026). Consequently, the technological objective of non-thermal processing is not simply to replace heat but to achieve an appropriate balance between microbial inactivation and preservation of the chemical, structural, nutritional, and functional characteristics of camel milk (Gebrehiwot & Banat, 2025; He et al., 2026; Mazumder et al., 2024; Mohamed et al., 2022).

Non-thermal processing encompasses several technologies that achieve microbial control through mechanisms other than conventional bulk heating, including HPP, PEF, ultrasonication, UV-C, cold plasma, and ozonation. Their effects, however, are highly dependent on processing intensity, treatment duration, temperature, microbial species, initial microbial load, and milk matrix characteristics, and their performance should not be generalized across technologies or experimental conditions. Thus, the designation “non-thermal” does not imply a complete absence of chemical or structural change: pressure can modify protein conformation, acoustic cavitation can alter protein structure and promote oxidation under severe conditions, and plasma and ozone can generate reactive species capable of modifying lipids, proteins, and vitamins. The following subsections therefore evaluate each technology according to its mechanism of action, demonstrated processing performance, camel-specific evidence, effects on camel milk quality, and current technological limitations, rather than treating these approaches as uniformly beneficial alternatives to pasteurization.

### 5.1 High-pressure processing

High-pressure processing is among the most extensively investigated non-thermal technologies for dairy preservation. The process subjects milk to hydrostatic pressure, commonly using water as the pressure-transmitting medium; commercial systems generally operate at pressures of up to approximately 600 MPa, although higher pressures can be applied at laboratory scale (Serna-Hernandez et al., 2021; Siddiqui et al., 2024; Urugo et al., 2023). Microbial inactivation during HPP is primarily associated with pressure-induced disruption of cellular membranes, alterations in membrane permeability and cellular morphology, interference with metabolic processes, and disruption of macromolecular and other essential cellular structures (Sehrawat et al., 2020). The extent of inactivation depends on pressure, treatment duration, temperature, initial microbial load, and the intrinsic resistance of the target microorganism (Kateh et al., 2024; Osaili et al., 2025; Sehrawat et al., 2021; Zagorska et al., 2021).

For camel milk, HPP is particularly attractive because it can provide substantial microbial reduction while limiting the direct thermal exposure associated with conventional pasteurization. Treatments in the range of 400–600 MPa have been investigated for achieving pasteurization-equivalent microbial safety under appropriate treatment conditions (Authority et al., 2021; Goyal et al., 2013; Singh et al., 2024). However, pasteurization-equivalent safety should not be interpreted as a universal outcome of applying 400–600 MPa: organism-specific log reductions and the corresponding pressure–time–temperature conditions must be considered, because different microorganisms exhibit different pressure resistance, and the initial microbial load and detection method also influence the apparent effectiveness of treatment (EFSA Panel on Biological Hazards et al., 2022; Kateh et al., 2024; Stratakos et al., 2019).

HPP can also alter the conformation and functional properties of milk proteins through pressure-induced changes in non-covalent interactions. Unlike thermal denaturation, which is strongly associated with disruption of hydrophobic interactions and exposure of reactive groups, pressure primarily affects molecular interactions involving volume changes and can therefore induce reversible or irreversible conformational changes depending on the pressure level and treatment duration (Grigera & McCarthy, 2010; Huppertz et al., 2004; López-Fandiño, 2006; Serna-Hernandez et al., 2021; Siddiqui et al., 2024). This may alter protein aggregation, solubility, gelation, and stability. Such effects are particularly relevant to camel milk because its distinctive protein composition and comparatively different thermal stability from bovine milk may influence its response to processing pressure (Ali et al., 2024; Ho et al., 2022). Reports of high retention of bioactive proteins following HPP should therefore be interpreted in relation to the specific proteins measured, the analytical method, the pressure–time–temperature conditions, and the milk species examined, rather than generalized to all bioactive components or processing conditions; pressure-induced structural modification is not necessarily equivalent to thermal denaturation, and the magnitude and reversibility of such changes depend on the specific protein and treatment combination examined.

Beyond microbial safety, HPP may reduce the need for chemical preservatives and can facilitate the production of minimally processed camel milk products with relatively good retention of fresh sensory characteristics (Osaili et al., 2025; Sehrawat, Kaur, Nema, Tewari and Kumar, 2020, Sehrawat, Kaur, Nema, Tewari and Kumar, 2021). Nevertheless, claims regarding environmental or sensory advantages should be considered in relation to the complete processing system, including energy requirements, equipment manufacture, throughput, packaging, and refrigeration, rather than the treatment step in isolation. The industrial potential of HPP is also influenced by factors beyond microbial efficacy: commercial operation commonly involves batch processing, which restricts throughput compared with continuous technologies and requires high capital investment and pressure-resistant packaging; bacterial spores, moreover, exhibit substantially greater pressure resistance than vegetative cells, and HPP does not eliminate the need for appropriate refrigerated storage and prevention of post-processing contamination (Rabbani et al., 2025; Saputra et al., 2025; Silva, 2019; Tomasula et al., 2014). Thus, although HPP currently has a relatively strong evidence base among emerging technologies for camel milk, its industrial readiness should be evaluated using microbial efficacy, quality retention, cost, throughput, scalability, packaging compatibility, regulatory requirements, and shelf-life, rather than microbial reduction alone.

### 5.2 Pulsed electric field processing

PEF processing applies short-duration, high-voltage electrical pulses to liquid foods. Its principal antimicrobial mechanism is electroporation, in which the applied electric field induces the formation of pores in microbial cell membranes, resulting in loss of membrane integrity and, at sufficient treatment intensity, cell death (Cavalcanti et al., 2023). PEF has attracted considerable interest for liquid foods, including dairy products, because it can operate continuously and with relatively limited thermal exposure, offering potential for preserving heat-sensitive nutritional, functional, and sensory attributes (Lytras et al., 2024; Siddique et al., 2025; Vashisht et al., 2024).

Despite this technological promise, direct experimental evidence for PEF application to camel milk remains substantially more limited than the evidence base for HPP and ultrasonication. Most available evidence derives from bovine and other dairy systems and provides useful mechanistic and technological context regarding electroporation rather than direct confirmation of performance in camel milk; differences in milk composition, electrical conductivity, fat content, and protein interactions may influence PEF performance in camel milk relative to these reference systems (Cavalcanti et al., 2023; Mohamad et al., 2023; Siddique et al., 2025; Vashisht et al., 2024). Accordingly, the potential of PEF for camel milk should be clearly distinguished from demonstrated camel-milk performance, and PEF should currently be considered a promising rather than an already-validated technology for this matrix.

Future camel milk studies should establish organism-specific log reductions under defined electric-field strengths, pulse widths and frequency, treatment duration, temperature, electrical conductivity, and initial microbial load. These experiments should also evaluate changes in protein structure, enzyme activity, vitamins, bioactive compounds, sensory quality, and shelf-life, to determine whether microbial benefits are achieved without unacceptable quality losses and before PEF can be reliably compared with HPP or conventional pasteurization for industrial camel milk processing.

### 5.3 Ultrasonication

Ultrasonication is an emerging technology for milk and dairy processing that uses high-frequency acoustic waves to generate cavitation. The collapse of acoustic bubbles produces localized high-energy conditions involving shear forces, pressure fluctuations, and microstreaming, which can disrupt microbial cells and modify the physical structure of milk components. Its relatively straightforward operation and potential compatibility with continuous liquid processing have contributed to increasing interest in dairy applications generally (Balthazar et al., 2019; Mudgil et al., 2022), and it has been investigated in camel milk specifically as an alternative or complementary approach to conventional heat treatment (Mudgil et al., 2022).

Recent evidence in camel milk demonstrated time-dependent microbial inactivation following ultrasonic treatment, with an approximately 2-log reduction after 5 min and reported complete bacterial inactivation after 15 min (El Kindoussy et al., 2026). These findings, however, require careful interpretation because the microbial species examined, initial microbial load, and analytical detection limit determine the significance of the reported reductions; culture-dependent non-detection should not automatically be interpreted as definitive evidence of complete microbial elimination, because injured or viable-but-nonculturable cells may remain undetected (El Kindoussy et al., 2026; Ganesan et al., 2015; Rathnakumar et al., 2024; Yu et al., 2026).

Beyond microbial control, ultrasonication can modify milk proteins and influence aggregation, solubility, interfacial properties, and the release or accessibility of bioactive peptides (Misra et al., 2017; Wali et al., 2020). In camel milk, the reported reduction in α-helix content accompanied by increased random-coil structures suggests partial protein unfolding associated with cavitation-induced mechanical effects, while relatively limited changes in overall physicochemical characteristics, mineral distribution, protein concentration, and lactose concentration suggest that treatment primarily induces physical and conformational rearrangement rather than extensive chemical degradation; ultrasound has additionally been reported to reduce particle size and turbidity and to modify zeta potential, indicating changes in colloidal organization (El Kindoussy et al., 2026). Such structural modification should not automatically be interpreted as beneficial or detrimental: its technological significance depends on whether it improves or compromises protein functionality, bioactivity, stability, and sensory properties. These effects can therefore be beneficial or detrimental depending on treatment severity moderate treatment may improve certain functional properties, whereas excessive acoustic intensity or prolonged exposure can increase structural disruption and potentially promote oxidative reactions so the optimal condition represents a balance between microbial reduction and preservation of protein and sensory quality rather than maximum acoustic intensity.

Ultrasonication has also been studied extensively in bovine, ovine, and other dairy systems, where effects on microbial stability, protein functionality, and bioactive peptide release have similarly been reported (Balthazar et al., 2019; D'Amico et al., 2006; Van Hekken et al., 2019; Wali et al., 2020). These studies provide useful comparative evidence, but their findings cannot be directly transferred to camel milk because differences in protein composition, mineral content, fat content, and colloidal structure may alter acoustic energy transfer and treatment response; the comparatively smaller camel milk evidence base therefore continues to limit direct conclusions regarding the relative efficacy and industrial readiness of ultrasonication for this matrix. Scale-up is a further consideration, because uniform energy distribution and control of treatment intensity can become more challenging as processing volume increases, and laboratory-scale treatment uniformity does not necessarily transfer to industrial scale. Consequently, further camel-specific studies under pilot- and industrial-scale conditions are required before laboratory-scale outcomes can be translated into commercial processing recommendations.

### 5.4 Ultraviolet radiation, cold plasma, and ozonation

UV-C, cold plasma, and ozonation represent promising emerging approaches, but their camel milk-specific evidence remains comparatively scarce. UV-C achieves microbial inactivation primarily through photochemical damage to microbial nucleic acids, particularly the formation of DNA lesions that interfere with replication (Delorme et al., 2020; Dhahir et al., 2021; Mazumder et al., 2024). However, the effectiveness of UV treatment in milk can be restricted by turbidity, fat content, suspended particles, and the optical properties of the milk matrix; consequently, UV dose alone cannot be used to predict microbial reduction without considering UV transmittance, flow configuration, microbial load, and treatment geometry (Christen et al., 2013; Delorme et al., 2020; Souroullas & Papademas, 2025).

Cold plasma generates reactive oxygen and nitrogen species that can damage microbial membranes, proteins, nucleic acids, and other cellular structures, while ozonation similarly relies on the strong oxidative capacity of ozone for microbial control. The same oxidative mechanisms responsible for microbial inactivation may, however, react with milk constituents: lipids are particularly susceptible to oxidation, while proteins, vitamins, pigments, and other bioactive compounds may also undergo oxidative modification under excessive treatment conditions (Bayati et al., 2024; Keewan et al., 2026; Nikmaram & Keener, 2022; Varga & Szigeti, 2016). The technological value of plasma and ozone should therefore be assessed by simultaneously measuring microbial reduction and indicators of lipid oxidation, protein modification, vitamin retention, antioxidant capacity, and sensory quality.

The limited number of camel-specific studies currently prevents firm conclusions regarding the optimum treatment intensity, microbial log reduction, or quality-retention capacity of UV-C, cold plasma, and ozonation in camel milk (Delorme et al., 2020; Dhahir et al., 2021). Evidence from other dairy and food systems can provide mechanistic guidance, but species- and matrix-specific validation is required before these technologies can be recommended for camel milk processing. This evidence gap is particularly important for plasma and ozone, because the chemical composition of camel milk may influence the generation, consumption, and reactivity of oxidative species.

### 5.5 Comparative technological perspective and research needs

The available evidence indicates that no single non-thermal technology can currently be considered universally superior for camel milk, and direct ranking of the technologies is not yet appropriate given the heterogeneity of processing conditions, microbial targets, analytical methods, and reported quality endpoints across studies. HPP currently has a stronger direct technological evidence base for camel milk, with comparatively robust evidence for microbial control and preservation of selected quality attributes; ultrasonication is supported by emerging camel-specific experimental evidence and has demonstrated potential for both microbial stabilization and modification of functional properties; PEF offers considerable potential for continuous processing but requires substantially more direct camel milk validation; and UV-C, cold plasma, and ozonation remain promising but are constrained by limited camel-specific evidence and, particularly for the oxidative technologies, the need to control potential chemical deterioration (Elsahoryi et al., 2026; Keewan et al., 2026; Pérez Núñez et al., 2024).

A meaningful comparison should therefore consider multiple performance criteria rather than microbial efficacy alone, including organism-specific microbial log reduction, retention of proteins and other bioactive compounds, effects on enzyme activity and physicochemical properties, sensory quality, energy and processing costs, throughput, scalability, packaging requirements, regulatory acceptance, process validation, post-treatment contamination, shelf-life, and cold-chain dependence. This broader framework is essential because a technology that achieves a high microbial reduction at laboratory scale may not necessarily provide the best overall outcome once quality retention, cost, throughput, and industrial implementation are taken into account. Assessment of post-treatment contamination and refrigerated shelf-life is likewise essential, because microbial inactivation during processing does not, by itself, guarantee microbiological safety throughout subsequent storage and distribution.

An important and recurring research gap is the scarcity of standardized, head-to-head comparisons of HPP, PEF, and ultrasonication using the same camel milk matrix, initial microbial load, target microorganisms, analytical methods, and quality indicators. Differences among existing studies in treatment intensity, milk composition, microbial load, analytical methods, and experimental scale make it difficult to establish universal dose–response relationships or definitive technology rankings, and instead risk conflating technologies that are merely promising with those that have demonstrated advantages under practically relevant conditions. Future research should therefore prioritize standardized comparative experiments that simultaneously quantify microbial reductions and chemical, biochemical, nutritional, and sensory responses across technologies. Such evidence would enable technology-specific processing windows to be established and would provide a stronger basis for determining which non-thermal approaches can progress from laboratory demonstration to reliable industrial application.

As shown in Fig. 2, each of the three technologies with the most direct camel-milk-relevant evidence base achieves microbial inactivation through a distinct primary mechanism acoustic cavitation for ultrasonication, isostatic pressure-induced membrane and protein disruption for HPP, and electroporation for PEF yet converges on a similar set of downstream benefits when appropriately optimized: reduced thermal degradation of heat-labile proteins, vitamins, and minerals; improved retention of bioactive peptides, immunoglobulins, and antioxidant compounds; and extension of microbiologically safe shelf life relative to untreated raw milk. This shared mechanism-to-outcome structure underscores why HPP, ultrasonication, and PEF, rather than UV-C, cold plasma, or ozonation, currently represent the most industrially advanced non-thermal options for camel milk, while also illustrating that none of them yet functions as a complete, standalone replacement for thermal pasteurization across every quality and safety dimension simultaneously.

**Fig. 2:**
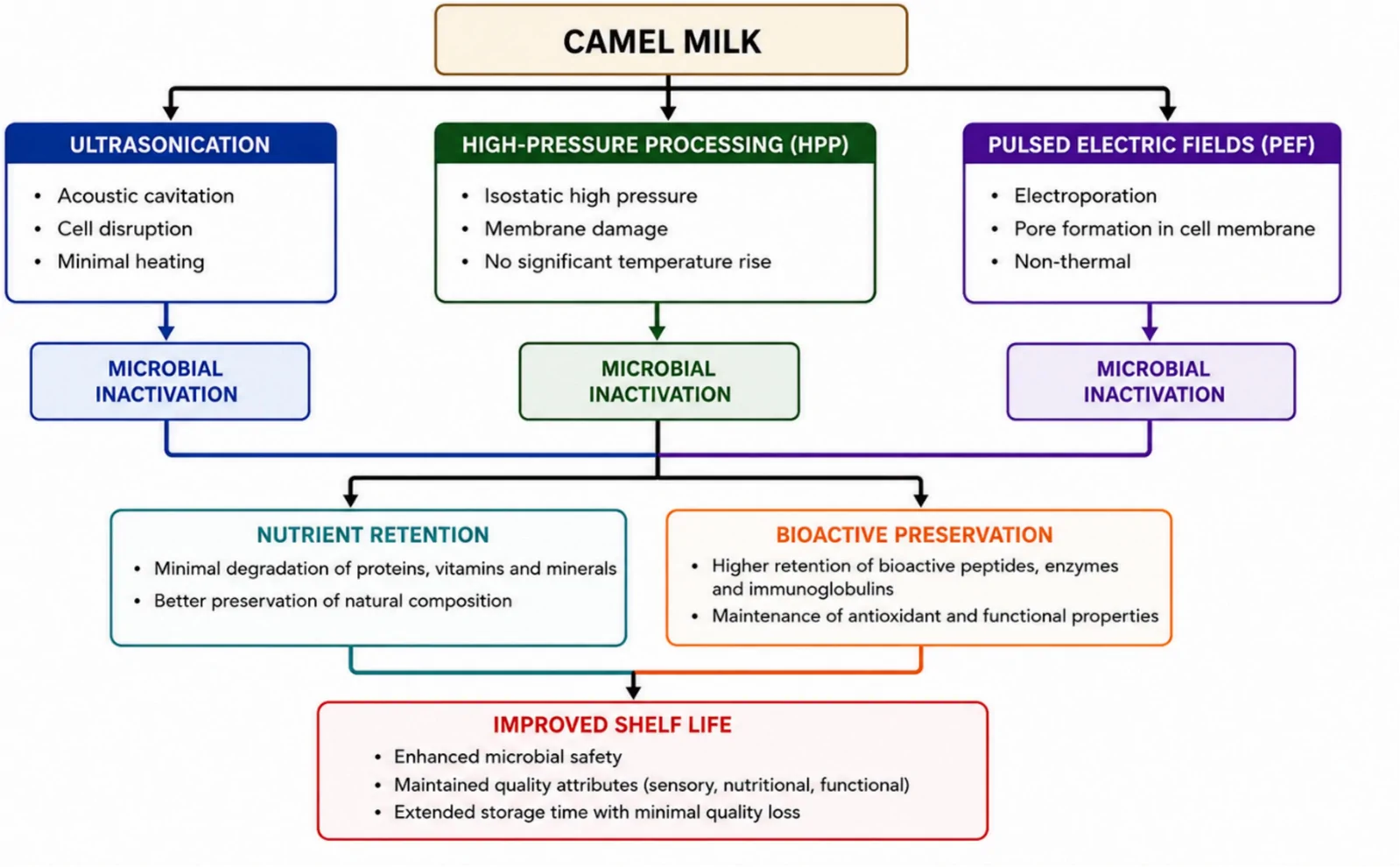
Summary of the three non-thermal technologies with the most direct camel-milk-relevant evidence base ultrasonication, HPP, and PEF situated within a common mechanistic and outcome framework

## 6 Impact on microbial safety of camel milk

Unprocessed camel milk can harbor a diverse range of spoilage and pathogenic microorganisms, with contamination levels varying substantially according to **geographical location, herd and animal health, milking hygiene, water quality, storage temperature, transportation practices, and time elapsed between milking and consumption.** For example, Abera et al. (2016) reported *Staphylococcus aureus*, *Streptococcus* spp., *Salmonella* spp., and *Escherichia coli* O157:H7 in 89.8%, 53.7%, 17.6%, and 31.5% of camel milk samples, respectively. **These percentages should not be interpreted as universal prevalence estimates for camel milk because they originated from a specific study population and geographical setting. Rather, they demonstrate the potential microbial hazards associated with raw camel milk under particular production and marketing conditions.** Similarly, studies in Egypt reported *S. enterica* and *E. coli* O157:H7 prevalences of 5% and 24%, respectively (Saeed et al., 2022), whereas *Listeria* spp. was detected in 14.3% of camel milk samples in Algeria (Lezzoum-Atek et al., 2023). **The variation among these estimates illustrates the importance of considering sample size, geographical location, sampling point, production system, and microbiological methods when comparing contamination data across studies.**

Total bacterial counts in raw camel milk have been reported to reach approximately 7 log CFU/mL, which can substantially reduce refrigerated shelf life (Mohamed & El Zubeir, 2014). High microbial loads generally reflect deficiencies in milking hygiene, animal health, equipment sanitation, storage temperature, and the duration between milking and processing (Hayes & Boor, 2001). **Contamination may also increase along the value chain, particularly when milk is transported and marketed without adequate temperature control. The detection of substantial microbial loads, including** *Salmonella***, at final marketing points therefore indicates that microbial safety should be considered across the entire camel milk value chain rather than only at the farm level (****Adugna et al.,** 2013**).**

**Importantly, contamination prevalence and processing efficacy represent different types of evidence. Prevalence studies establish the occurrence and distribution of microbial hazards in raw camel milk, but they do not demonstrate whether a particular non-thermal technology can eliminate those hazards. Conversely, processing experiments quantify microbial reductions under defined treatment conditions but may use artificially inoculated milk, laboratory-scale systems, or relatively low initial microbial loads. Therefore, contamination surveys should be used to define the relevant microbial hazards and inform process-validation targets, rather than being interpreted as direct evidence of the effectiveness of a non-thermal treatment.**

### 6.1 Linking microbial hazards with non-thermal processing efficacy

**The practical value of a non-thermal technology depends on its ability to achieve an adequate reduction of the microorganisms relevant to the specific camel milk production system. This requires consideration of organism-specific resistance rather than assuming that a single treatment condition provides equivalent inactivation across pathogens.** Vegetative bacteria such as *E. coli*, *Salmonella*, *Staphylococcus*, and *Streptococcus* spp. may respond differently to pressure, acoustic cavitation, electric fields, ultraviolet radiation, plasma, or ozone. **Accordingly, process validation should report quantitative** log **reductions together with the target organism, initial microbial concentration, treatment intensity, treatment duration, temperature, and analytical detection limit.**

For HPP, for example, microbial inactivation is influenced by pressure, holding time, temperature, microbial physiology, and food-matrix effects. **Thus, the commonly reported pressure range of 400–600 MPa cannot itself be regarded as a defined microbial safety target. A validated HPP process should demonstrate the required** log **reduction for the relevant microorganisms under specified pressure-time-temperature conditions.** Similarly, ultrasonic treatment has been reported to produce approximately a 2-log reduction after 5 min and reported complete inactivation after 15 min in camel milk (El Kindoussy et al., 2026). **These findings should be interpreted in the context of the microorganisms examined, initial microbial load, ultrasound intensity and frequency, treatment temperature, and detection method. In particular, culture-dependent non-detection does not necessarily demonstrate complete microbial elimination because injured cells, viable-but-nonculturable cells, or highly resistant subpopulations may remain undetected.**

**PEF, UV-C, cold plasma, and ozonation require similar organism-specific validation. For PEF, microbial reduction depends on electric-field strength, pulse characteristics, treatment time, and conductivity of the milk matrix. UV-C efficacy is influenced by UV transmittance and optical path length, while plasma and ozone depend on the concentration and reactivity of generated oxidizing species. Consequently, treatment parameters should be linked explicitly to quantitative microbial reductions rather than described simply as “effective” or “promising.”**

### 6.2 Evidence base and limitations

**The current evidence base is heterogeneous. HPP and ultrasonication have comparatively more direct camel milk evidence, whereas PEF, cold plasma, UV-C, and ozonation remain less extensively validated in camel milk. Some studies included in the literature provide direct experimental evidence from camel milk, whereas others are reviews or experiments conducted in bovine or other dairy systems. Evidence from non-camel dairy systems is useful for understanding mechanisms and technological feasibility but should not be interpreted as direct evidence of performance in camel milk.** This distinction is particularly important when evaluating technologies for industrial application because differences in milk composition, turbidity, electrical conductivity, protein structure, and microbial ecology may influence treatment efficacy.

Table 1 **summarizes the available information on quantitative processing conditions and microbial outcomes. The table also highlights the principal quality endpoints and limitations of the available evidence.** This classification helps avoid overestimating the maturity of technologies for camel milk and emphasizes where additional validation is required.

**Table 1:** Summary of studies investigating non-thermal processing technologies for camel milk preservation and quality enhancement.

Technology	Study (author, year, source) & evidence type	Camel breed / origin & sample size	Quantitative processing conditions	Microbial outcome	Quality / functional outcome	Evidence limitation
**Ultrasonication**	Mudgil et al. (2022), Original camel-milk experiment (with bovine-milk comparator)	UAE (Al Ain); breed not stated. *n* = 4 replicate 100 mL samples per treatment; camel and bovine milk run in parallel over 7 d storage at 4 °C.	6 discrete ultrasound power levels (US1–US6, ∼100–210 W), benchmarked against flash-heat pasteurization (FHP).	≥140 W (US3): ∼4-log reduction in bacterial load; complete inactivation at 170 W (US5) and 210 W (US6). Significance testing reported in source; exact *P*-values not stated.	No significant change in protein electrophoretic pattern with either US or FHP.	Breed/origin and exact per-day sample size not detailed; single-site (UAE) source.
**Ultrasonication**	El Kindoussy et al. (2026), Original camel-milk experiment	Morocco; *Camelus dromedarius* (breed not further specified). Exact sample size and frequency/power not stated.	Controlled ultrasonic cavitation; time-course assessed at 5 min and 15 min endpoints.	∼2-log reduction at 5 min; complete (culture-based, non-detect) bacterial inactivation at 15 min.	No significant change in overall physicochemical properties; reduced particle size and turbidity; altered zeta potential; reduced α-helix / increased random-coil content (partial protein unfolding).	Species/detection-limit basis for “complete inactivation” not specified; frequency/amplitude values.
**HPP**	Aljasass et al., (2023), Original camel-milk experiment	Saudi Arabia; breed and exact sample size is not stated	200–600 MPa, 5 min, 40 °C.	200 MPa: 0.12-log reduction; up to 400 MPa: >3-log reduction. Gram-negative bacteria more pressure-sensitive than Gram-positive; Enterobacteriaceae controlled at ≥300 MPa.	Pressure ≥ 350 MPa induced clotting of camel milk a camel-specific vulnerability not observed in cow or goat milk, nor in heat-treated camel milk.	Sample size and breed not reported.
**HPP**	Osaili et al. (2025), Original camel-milk experiment.	UAE; breed not stated, sample size-13 batches, each containing 6 × 50-mL lots.	350 MPa for L. *monocytogenes* and *Salmonella*; 300 MPa for E. coli O157:H7, at 10 °C for 1, 2, 3, 4, and 5 min. Storage after HPP- 4 °C and 10 °C, for 0, 1, 4, 7 and 10 days	Initial total plate count 3.93 ± 0.41 log CFU/mL (4 °C) and 3.52 ± 0.29 log CFU/mL (10 °C); lactic-acid-bacteria population tracked across treatment × storage combinations. *p* < 0.05, Tukey-type compact-letter display (a–g) used for mean separation.	Not detailed beyond microbiological endpoints. HPP at 350 MPa for up to 5 min did not significantly alter proteolytic activity or the apparent casein micelle/protein profile.	Single farm and unreported breed; limited biological representativeness; artificial pathogen inoculation; relatively narrow principal pressure range; limited quality endpoints; only 10-day storage; background microbiota showed limited reduction.
**PEF**	Mazumder, Ali & Banat (2024), Review (camel-milk-focused synthesis; not primary PEF data).	N/A- secondary literature.	N/A- synthesizes conditions reported in primary literature; no conditions independently generated.	Synthesizes reported potential for microbial inactivation across non-thermal approaches, including PEF, for camel milk.	Synthesizes reported potential for quality/bioactive retention across approaches.	Non-primary evidence; the review itself notes that genuine camel-specific PEF primary data remain scarce.
**Cold plasma**	Keewan et al. (2026), Original camel-milk experiment.	UAE, breed and exact sample size is not stated	Central composite design evaluating gas composition (air, M65, N₂), voltage (33–42 kV), electrode gap (20–40 mm), and treatment duration. Optimized treatment: M65 gas (65% O₂ + 30% CO₂ + 5% N₂), 42 kV, 40-mm electrode gap, 15 min. Milk surface temperature remained <29 °C, supporting non-thermal operation.	Approximately 3-log / >99.9% reduction in total bacterial count under optimized DBD-CP conditions. The study evaluated natural microflora, rather than experimentally inoculated pathogens.	pH and freezing point were not significantly altered. Lipid oxidation remained very low (TBARS ≤0.17 mg MDA/kg). Color change was small (ΔE ≈ 1.94) and below the reported perceptible threshold. Fatty-acid profile and lipid stability were maintained. Sensory evaluation indicated minimal changes in flavor and odor.	Single-herd/bench-scale study, with breed and exact sample size not reported; natural microflora rather than specific pathogens; immediate post-treatment quality assessment; no comprehensive storage validation; no toxicological assessment; pathogen-specific inactivation was not established.
**Ultraviolet-C (UV-C)**	Dhahir et al. (2021), Original camel-milk experiment.	USA-sourced camel milk (breed and sample size not stated); Camel milk; pasteurized and experimentally inoculated with *E. coli* O157:H7 and *Salmonella typhimurium*; continuous-flow laboratory system	UV-C 4.15, 8.30 and 12.45 mJ/cm^2^ UV-C doses in a continuous-flow system	At 4.15, 8.30 and 12.45 mJ/cm^2^, *E. coli* O157:H7 reductions were 1.9, 3.3 and 3.9 log, respectively; *S. typhimurium* reductions were 0.9, 3.0 and 3.9 log, respectively.	TBARS/lipid peroxidation: no significant differences. Protein profiles: no significant changes in αs1-casein, α-lactalbumin or lactoferrin. However, cis-9,trans-11 CLA decreased and trans-10,cis-12 CLA increased after UV-C treatment.	5-log FDA pathogen-reduction target was not achieved, even at 12.45 mJ/cm^2^. Breed and sample size was not reported; milk was experimentally inoculated and therefore may not represent naturally contaminated milk; continuous-flow conditions may differ from commercial systems; limited doses were evaluated; functional/bioactive properties were not comprehensively assessed; longer-term storage and shelf-life effects were not established.
**Electromagnetic / microwave-assisted processing**	Mazumder et al. (2026), Original camel-milk experiment.	UAE; breed and exact sample size are not stated.	Electromagnetic field (EM): 100, 200, 400, 600, 800 and 850 mT, with the optimized treatment at 850 mT for 3 min. Microwave (MW): 250 W for 30 s. TCM was used as a benchmark.	Microbial reduction increased with EM field strength: 100 mT: 0.05 log; 200 mT: 1.03 log; 400 mT: 1.30 log; 600 mT: 2.39 log; 800 mT: 2.53 log. At the optimized 850 mT/3 min, the microbial load decreased from 6.8 × 10^6^ to 1.5 × 10^4^ CFU/mL, equivalent to 2.66-log reduction (99.78%). Both EM and MW extended refrigerated shelf life.	EM treatment showed better retention of protein, carbohydrates, minerals and other nutritional components than MW and thermal pasteurization. Structural analyses indicated minimal protein denaturation and oxidative damage under EM treatment. MW caused greater nutrient losses than EM. EM better preserved immune-active/bioactive proteins than MW and thermal pasteurization. Antioxidant capacity (DPPH) was reported as 62.4 ± 2.1% in raw milk, 60.8 ± 1.8% after EM, 54.2 ± 2.5% after thermal pasteurization, and 49.7 ± 2.2% after MW. Sensory acceptability favored EM treatment (8.0 ± 0.65).	Breed and sample size were not reported; limited information available on the number of independent camel/milk sources; microbial challenge was not pathogen-specific; the approximately 2.66-log reduction at the optimum EM condition does not establish 5-log pasteurization-equivalent pathogen inactivation; shelf-life findings require independent validation; scale-up and industrial process validation remain necessary.

## 7 Impact on nutritional and bioactive compounds of camel milk

Camel milk contains a distinctive combination of proteins, essential amino acids, vitamins, minerals, unsaturated fatty acids, and bioactive components, including lactoferrin, lysozyme, immunoglobulins, lactoperoxidase, and bioactive peptides (Alhassani, 2024; Arain et al., 2022; Behrouz et al., 2022; Khaliq et al., 2024; Swelum et al., 2021). **The significance of these constituents in the context of non-thermal processing lies not simply in their presence but in their differential sensitivity to processing-induced structural and chemical changes. Thus, preservation of nutritional and bioactive quality should be evaluated at the level of individual compounds and functional activities rather than by total protein or general compositional measurements alone.**

The composition of camel milk varies with breed, diet, lactation stage, geographical origin, season, and animal management (Alhassani, 2024; Mohammed & Alshaibani, 2024; Swelum et al., 2021; Wang et al., 2024). **This inherent variability can influence the response of milk to processing and partly explains differences among studies reporting retention or degradation of individual components. Consequently, comparisons among processing technologies should account for differences in the initial concentration of target compounds, analytical methods, treatment conditions, and storage period.**

### 7.1 Nutritional composition

Camel milk provides high-quality proteins containing essential amino acids, unsaturated fatty acids, vitamins, minerals, and other nutrients (Alhassani, 2024; Arain et al., 2022; Khaliq et al., 2024; Swelum et al., 2021). It is particularly recognized for its relatively high vitamin C content compared with bovine milk and its distinctive mineral and protein composition (Alhassani, 2024; Bakry et al., 2021). **However, the nutritional value of these constituents after processing depends on their chemical and structural stability. Proteins may undergo unfolding, aggregation, hydrolysis, or chemical modification, while vitamins may be lost through oxidation or thermal degradation. Minerals are generally more chemically stable than proteins and vitamins, but their apparent distribution between soluble and colloidal phases may change as a consequence of processing-induced alterations in protein and micellar structure.**

**Non-thermal processing may reduce some of these changes by limiting exposure to elevated temperatures, but preservation is technology- and treatment-dependent. HPP can induce pressure-dependent protein conformational changes without necessarily producing the same structural modifications as conventional heating, whereas ultrasonication can alter protein conformation and colloidal organization through cavitation. PEF has potential for minimizing thermal damage in liquid foods, but direct evidence for nutrient retention in camel milk remains limited.**

### 7.2 Bioactive compounds and their processing stability

Camel milk contains multiple classes of bioactive compounds, including bioactive peptides, lactoferrin, lysozyme, immunoglobulins, lactoperoxidase, and other protein-associated components (Behrouz et al., 2022; Dharmisthaben et al., 2021; Liu et al., 2023; Mohottige et al., 2024; Swelum et al., 2021). Bioactive peptides may exhibit antioxidant, ACE-inhibitory, antimicrobial, and enzyme-inhibitory activities, depending on their amino acid sequence and molecular structure. **Importantly, processing may either preserve these activities or modify them through protein unfolding, aggregation, hydrolysis, oxidation, or changes in peptide accessibility. Thus, the effect of processing cannot be inferred solely from the measured concentration of total protein or peptide content.**

**Lactoferrin, immunoglobulins, lysozyme, and lactoperoxidase are particularly relevant because their biological functions depend on preservation of specific molecular structures. Severe heat exposure can reduce their functional activity through denaturation or structural modification, whereas milder or non-thermal treatments may provide greater retention. Nevertheless, the available literature does not yet provide sufficient standardized quantitative comparisons of the retention of all four proteins across HPP, PEF, ultrasonication, UV-C, cold plasma, ozonation, and conventional pasteurization. This represents an important evidence gap rather than justification for assuming that one non-thermal technology universally provides superior bioactive retention.**

**The response of bioactive compounds to ultrasonication is similarly treatment-dependent. Moderate acoustic treatment may alter protein conformation and facilitate the release or accessibility of bioactive peptides, whereas excessive treatment may promote structural disruption or oxidative reactions. In camel milk,** **El Kindoussy et al. (**2026**)** **reported changes in protein secondary structure following ultrasound treatment, including reduced α-helix and increased random-coil content, indicating partial protein unfolding. Such structural changes should be evaluated alongside biological activity to determine whether they represent functional enhancement, preservation, or degradation.**

Recent evidence further strengthens the specific link between camel-derived peptides and metabolic activity. **Lin et al. (2026) identified novel α-glucosidase-inhibitory peptides from Bactrian camel milk, providing direct evidence that camel-derived peptides can exert enzyme-inhibitory activity relevant to postprandial glucose regulation. This finding provides a more specific biochemical basis for the antidiabetic potential of camel milk than broad claims based solely on its overall nutritional composition. It also highlights the importance of determining whether processing preserves, destroys, or generates such bioactive peptide sequences.**

### 7.3 Effects of non-thermal processing on bioactive integrity

**The principal advantage of non-thermal processing in relation to bioactive integrity is the potential to reduce the thermal burden while maintaining microbial safety. However, the available evidence indicates that retention varies among compounds and technologies. HPP may preserve selected proteins and functional properties under appropriately controlled conditions, although pressure-induced conformational changes can occur. Ultrasonication can maintain selected physicochemical characteristics while simultaneously modifying protein structure. PEF offers potential for preservation because of its limited thermal exposure, but direct camel milk evidence remains insufficient. UV-C, cold plasma, and ozonation require additional attention because their mechanisms involve radiation or reactive species that may affect sensitive proteins, lipids, vitamins, and other bioactive constituents.**

**Particular caution is required for oxidative technologies. Reactive oxygen and nitrogen species generated during cold plasma treatment, and the strong oxidative capacity of ozone, may contribute to microbial inactivation but can also promote lipid oxidation and oxidative modification of proteins and vitamins if treatment conditions are excessive. Therefore, assessment of these technologies should include not only microbial reductions but also indicators of lipid oxidation, protein integrity, antioxidant capacity, vitamin retention, and sensory quality.**

Fermentation represents a related processing approach that may increase the diversity and accessibility of bioactive peptides through microbial proteolysis (Bhuva et al., 2025; Dharmisthaben et al., 2021). **However, fermentation should not be considered equivalent to non-thermal preservation because it involves biochemical transformation of the milk matrix. Its relevance to this review is primarily as evidence that processing can modify bioactivity either by preserving existing compounds or by generating new bioactive peptides.** Heat treatment and storage can similarly alter protein structure and bioactive activity, with the magnitude of change dependent on treatment severity and duration (Su et al., 2025; Wang et al., 2024).

### 7.4 Health-related functional properties and evidence limitations

Camel milk bioactive compounds have been associated with antioxidant, antimicrobial, anti-inflammatory, antihypertensive, antidiabetic, immunomodulatory, and other biological activities (Behrouz et al., 2022; Izadi et al., 2019; Swelum et al., 2021). **However, these biological activities should be distinguished from established clinical therapeutic effects. Evidence for antidiabetic activity, for example, includes biochemical studies, animal experiments, and clinical investigations of varying size and methodological quality. The identification of α-glucosidase-inhibitory peptides by Lin et al. (2026) provides mechanistic evidence, but it does not by itself establish a clinical antidiabetic effect in humans.**

Similarly, reported associations with cancer, autism, liver and kidney disorders, metabolic syndrome, and other diseases should be interpreted cautiously. **Most evidence remains preclinical or is derived from relatively small clinical studies, and processing-specific evidence linking preservation of individual bioactive compounds to clinical outcomes is particularly limited. Accordingly, this review does not consider retention of a bioactive compound during processing to constitute evidence of therapeutic efficacy.**

**The critical processing question is therefore whether non-thermal technologies can maintain the molecular integrity and biological activity of nutritionally and functionally important compounds while achieving an appropriate level of microbial safety. Addressing this question requires compound-specific measurements rather than generalized statements of “bioactive retention.” Future studies should quantitatively compare lactoferrin, immunoglobulins, lysozyme, lactoperoxidase, vitamins, antioxidant activity, and relevant bioactive peptides before and after each processing technology, using conventional pasteurization as an appropriate comparator where feasible.**

**Overall, the available evidence supports the potential of non-thermal processing to improve the balance between microbial safety and preservation of camel milk quality, but the strength of evidence differs substantially among technologies and compounds. HPP currently provides relatively stronger evidence for preservation of selected quality attributes, whereas evidence for PEF, UV-C, cold plasma, and ozonation remains comparatively limited in camel milk. Establishing compound-specific retention, structural integrity, biological activity, and dose-response relationships under standardized processing conditions will be essential for determining whether the apparent advantages of non-thermal technologies translate into meaningful improvements in the nutritional and functional quality of camel milk.**

## 8 Challenges and limitations of camel milk

Despite the potential of non-thermal technologies to improve the safety and quality of camel milk, their translation from experimental applications to routine industrial use remains constrained by **economic, infrastructural, technological, regulatory, and evidence-related limitations.** These constraints are particularly important in camel-producing regions, where processing systems frequently operate at small or medium scale and under geographically dispersed production conditions.

### 8.1 Economic and infrastructural constraints

A major barrier is the capital and operational cost associated with technologies such as HPP and PEF, which require specialized equipment, maintenance, trained personnel, and appropriate processing facilities (Neokleous et al., 2022). **Although these costs may be justified in high-throughput centralized facilities, they can be difficult to recover in small-scale camel milk enterprises where production volumes are seasonal and geographically dispersed.** HPP systems are particularly challenging because of their batch-oriented operation and substantial equipment requirements (Sehrawat et al., 2020). **Therefore, technology selection should consider throughput, utilization rate, product value, energy demand, maintenance requirements, and the scale of operation rather than capital cost alone.**

Infrastructure presents a related constraint. Much camel milk is produced in remote arid and semi-arid regions where reliable electricity, refrigeration, transportation, and processing facilities may be limited (Konuspayeva & Faye, 2021; Seifu, 2023). **This is particularly relevant for technologies such as PEF and cold plasma that require stable electrical power, while all preservation approaches remain dependent on appropriate post-treatment storage. The benefit of microbial inactivation can be substantially reduced if treated milk is subsequently exposed to inadequate refrigeration, prolonged transportation, or post-processing contamination.** Thus, implementation cannot be evaluated independently of the broader cold-chain and distribution system (Adugna et al., 2013).

### 8.2 Suitability for decentralized and pastoral production systems

The characteristics of camel production create additional challenges for conventional centralized processing models. Camel herding is often practiced under conditions of seasonal mobility, dispersed settlements, extreme temperatures, and limited access to water and electricity (Sazmand & Nourian, 2023). **Consequently, technologies developed primarily for stationary, high-throughput dairy plants may require adaptation before they can be effectively deployed close to production sites. HPP, for example, uses a pressure-transmitting liquid and is generally operated in batch systems, which can constrain its application in highly decentralized settings (****Naveena & Nagaraju,** 2020**).**

**Potential solutions should therefore focus on context-appropriate implementation rather than assuming that the same processing configuration is suitable across all camel-producing regions. Small-scale modular systems, shared processing facilities, mobile collection and processing units, and renewable-energy-supported equipment may offer potential pathways for improving accessibility. However, these approaches should currently be regarded as implementation options requiring technical and economic validation rather than established solutions.** Their feasibility will depend on processing capacity, mobility, energy requirements, water availability, maintenance infrastructure, and integration with existing milk-collection systems.

### 8.3 Scientific and process-validation gaps

A major scientific limitation is the relatively small body of camel-specific evidence compared with the extensive literature available for bovine milk. **Evidence from bovine and other dairy systems provides useful mechanistic and technological information, but direct extrapolation is inappropriate without validation because camel milk differs in protein composition, casein micelle structure, mineral distribution, and physicochemical properties (****Ho et al.,** 2022**;** **Lajnaf et al.,** 2022**).** These differences may affect microbial inactivation and the stability of proteins, lipids, vitamins, and bioactive compounds during HPP, PEF, ultrasonication, plasma, UV-C, or ozonation.

**A particularly important gap is the limited availability of organism-specific dose-response data in camel milk. Process validation requires quantitative relationships between treatment intensity, exposure time, temperature, milk composition, initial microbial load, and microbial reduction. Such information is necessary to establish scientifically defensible critical process parameters and to determine whether a technology can consistently achieve the required level of safety.** Greater emphasis is also needed on surrogate selection, resistant microorganisms, microbial recovery methods, and the potential survival of injured or viable-but-nonculturable cells following treatment.

### 8.4 Regulatory, validation, and post-processing considerations

**Industrial readiness extends beyond demonstrating microbial reduction under laboratory conditions. Regulatory acceptance requires validated processes, appropriate pathogen-surrogate selection, defined critical limits, reproducible microbial reductions, and evidence that the treatment remains effective under realistic production conditions. In addition, post-treatment contamination, packaging integrity, refrigerated shelf life, and storage and distribution conditions must be incorporated into process validation.** These considerations are particularly important for camel milk because improvements achieved during processing may be compromised by subsequent handling under weak cold-chain conditions.

**The limited availability of standardized camel-specific validation data also complicates regulatory assessment and comparison among technologies. Future research should therefore move beyond reporting whether a technology is “effective” and instead establish quantitative performance criteria that integrate microbial safety, quality retention, throughput, energy consumption, cost, and scalability.**

### 8.5 Overall implementation perspective

**The principal challenge is therefore not the absence of technically promising technologies but the gap between laboratory-scale performance and context-appropriate implementation. HPP, PEF, ultrasonication, cold plasma, UV-C, and ozonation differ substantially in equipment requirements, operating conditions, scalability, and maturity of camel-specific evidence. Consequently, no single technology can currently be considered universally optimal for camel milk.** Future implementation should prioritize **technology-specific validation, decentralized processing models where appropriate, integration with reliable cold-chain systems, and techno-economic and life-cycle assessments under representative camel-producing conditions.** Such an approach would help distinguish technologies that are experimentally promising from those that are genuinely feasible for sustainable camel milk processing.

## 9 Future perspectives and research needs

Future development of non-thermal processing for camel milk should move from demonstrating technological feasibility toward establishing validated, scalable, and context-appropriate processing systems. Priority should therefore be given to research that integrates microbial safety, preservation of nutritional and bioactive quality, processing cost, energy demand, throughput, scalability, and regulatory requirements. Rather than assuming that emerging technologies are inherently suitable for pastoral production, their feasibility should be demonstrated under realistic camel milk production and distribution conditions.

Solar energy represents a potentially relevant option for addressing the electricity constraints encountered in many camel-producing regions. Photovoltaic systems could potentially support electricity-dependent technologies such as ultrasonication, PEF, UV-C, and cold plasma, as well as refrigeration required after processing. However, these applications currently represent promising concepts rather than demonstrated solutions for camel milk processing. Future studies should therefore quantify energy requirements, processing capacity, battery-storage requirements, equipment performance under variable solar availability, maintenance needs, and overall operating costs. Techno-economic and life-cycle assessments are particularly needed to determine whether solar-integrated systems provide practical environmental and economic advantages compared with conventional electricity-based processing.

Low-cost and decentralized processing configurations also warrant investigation, particularly for small-scale producers and dispersed pastoral communities. Potential approaches include compact ultrasonication or UV-C systems, modular PEF units, and appropriately designed atmospheric-pressure plasma systems. However, reducing equipment size or cost should not compromise process validation or microbial safety. Each proposed adaptation should therefore be evaluated against defined microbial log-reduction targets, treatment uniformity, energy consumption, maintenance requirements, and the ability to operate under field conditions. HPP may remain more appropriate for centralized processing facilities because of its equipment requirements and batch operation, whereas smaller-footprint technologies may offer greater potential for decentralized applications.

A major research priority is the generation of camel-specific process-response relationships. Future studies should establish organism-specific inactivation kinetics for relevant pathogens and spoilage microorganisms while simultaneously measuring changes in lactoferrin, immunoglobulins, lysozyme, lactoperoxidase, vitamins, antioxidant activity, and other relevant bioactive compounds. Studies should report standardized processing parameters, initial microbial loads, log reductions, detection limits, physicochemical changes, sensory outcomes, and refrigerated shelf life to enable meaningful comparison among technologies. Particular attention should be given to potential oxidative effects associated with plasma and ozonation and to structural changes induced by pressure and ultrasound.

Research should also distinguish demonstrated technological performance from expected applicability. PEF, cold plasma, UV-C, and ozonation remain comparatively underexplored in camel milk, and evidence from bovine or other dairy systems should be used primarily to formulate hypotheses and guide experimental design rather than to establish camel-specific efficacy. Similarly, proposed mobile processing units, solar-powered systems, and community-level processing models should be subjected to field validation before being considered implementation-ready.

Finally, future work should incorporate regulatory and supply-chain dimensions alongside technological development. Process validation should address pathogen-surrogate selection, critical process parameters, post-treatment contamination, packaging, cold-chain dependence, and shelf-life under realistic distribution conditions. Techno-economic analysis, life-cycle assessment, and pilot-scale trials in representative camel-producing regions are particularly important for determining whether laboratory-scale advances can be translated into economically viable processing systems. Establishing standardized camel-specific evidence and aligning it with national food-safety regulations will ultimately be necessary to support regulatory acceptance, industry investment, consumer confidence, and sustainable expansion of formal camel milk markets.

## 10 Conclusion

Camel milk possesses a distinctive nutritional and bioactive profile, but its physicochemical stability and functional quality can be affected by conventional thermal processing. The evidence reviewed indicates that non-thermal technologies, particularly HPP and ultrasonication, have demonstrated promising potential for achieving microbial control while reducing thermal damage to proteins and other quality-related constituents. PEF, UV-C, cold plasma, and ozonation also offer potential advantages, although camel-specific experimental evidence remains comparatively limited. Importantly, the effectiveness of these technologies is highly dependent on treatment intensity, time, temperature, milk composition, target microorganisms, and processing configuration; therefore, non-thermal processing should not be regarded as inherently superior or universally capable of preserving all bioactive compounds.

The transition from laboratory-scale evidence to industrial application remains constrained by equipment costs, energy and infrastructure requirements, limited camel-specific microbial inactivation and bioactive-retention data, regulatory uncertainties, and dependence on appropriate post-processing cold-chain conditions. Future research should prioritize standardized, organism-specific process validation; quantitative assessment of individual bioactive compounds and functional activity; comparative evaluation against conventional pasteurization; and techno-economic and life-cycle assessments under realistic camel-producing conditions. Development of context-appropriate processing models, including decentralized or renewable-energy-supported systems, should be guided by demonstrated feasibility rather than conceptual potential. Addressing these gaps will be essential for translating the promising technological performance of non-thermal processing into safe, scalable, economically viable, and quality-preserving camel milk processing systems.

## CRediT authorship contribution statement

**Shamsedin Mahdi Hassan:** Writing – review & editing, Writing – original draft, Formal analysis, Data curation, Conceptualization. **Tilahun Abera Teka:** Writing – review & editing, Writing – original draft, Formal analysis, Data curation, Conceptualization. **Samatar Abshir Mahamed:** Writing – review & editing, Writing – original draft, Formal analysis, Data curation, Conceptualization. **Maslah Mahamed Duale:** Writing – review & editing, Data curation, Conceptualization. **Abdurazak Tahir Awale:** Methodology, Validation, Visualization, Writing – original draft, Writing – review & editing. **Markos Makiso Urugo:** Writing – review & editing, Writing – original draft.

## Declaration of competing interest

The authors declare that they have no known competing financial interests or personal relationships that could have appeared to influence the work reported in this paper.

## Data Availability

The data that has been used is confidential.
